# Walking to the Beat of Their Own Drum: How Children and Adults Meet Timing Constraints

**DOI:** 10.1371/journal.pone.0127894

**Published:** 2015-05-26

**Authors:** Simone V. Gill

**Affiliations:** Boston University, Departments of Occupational Therapy and Medicine, Program in Rehabilitation Sciences (PhD), Boston, Massachusetts, United States of America; National University of Singapore, SINGAPORE

## Abstract

Walking requires adapting to meet task constraints. Between 5- and 7-years old, children’s walking approximates adult walking without constraints. To examine how children and adults adapt to meet timing constraints, 57 5- to 7-year olds and 20 adults walked to slow and fast audio metronome paces. Both children and adults modified their walking. However, at the slow pace, children had more trouble matching the metronome compared to adults. The youngest children’s walking patterns deviated most from the slow metronome pace, and practice improved their performance. Five-year olds were the only group that did not display carryover effects to the metronome paces. Findings are discussed in relation to what contributes to the development of adaptation in children.

## Introduction

Everyday walking requires adapting gait to meet task constraints. However, to adapt walking in the face of constraints, walkers need to obtain several abilities. First, they must acquire skilled walking abilities. Second, walkers need to harness that skill to modify walking in the moment. For example, changing direction, slowing down, or speeding up to navigate through the environment requires exploiting skilled walking in accordance with the circumstances at hand [1, 2].

Modifying walking to meet task constraints can pose challenges for children. Specifically, children demonstrate skill in walking [3], but they exhibit difficulty with altering their walking to meet constraints. Between 5- to 7-years old, the distance and timing of school-aged children’s steps match the consistency and coordination observed in adult walking [4–6]. In contrast, compared to adults, children have more trouble modifying their walking to meet constraints [7, 8]. The effect also seems to be greater in younger versus older children. For example, when asked to synchronize clapping and walking at self-selected paces, 6- to 8-year old children demonstrate more consistency than 4-year-olds [7]. If an audio metronome is introduced during the same task, 4-year-olds’ movements are more variable compared to 6-, 8-, and 10-year olds as well as adults. Six- and 8-year olds do not differ from one another in terms of movement variability, but are more variable than 10-year-olds and adults [8]. Children’s attempts to meet constraints can result in decreased accuracy. Compared to 7- to 12-year olds, 4- to 6-year old children modify their walking when carrying a box by taking shorter steps, but have difficulty keeping the box level [9].

Despite needing to constantly adapt to meet constraints, there is limited knowledge about 5- to 7-year olds’ ability to modify their walking in the face of constraints. We also know little about how their attempts to modify gait would compare to that of adults and whether practice would influence their ability to adapt their gait. Understanding whether children can adapt their gait is particularly important during the early school years when children are introduced to activities that require coordinating ongoing actions to meet constraints. For instance, children must meet timing constraints when walking in line at school; children need to speed up or slow down their steps to keep pace with others.

The purpose of the current study was to depict school-aged children’s walking modifications as they attempted to walk to imposed metronome paces. A walking task was constructed in which 5- to 7-year-old children and adults walked to slow and fast metronome paces. The paces were 30% slower and 15% faster than participants’ average cadences (steps per minute). Walking strategies used to adapt to the metronome paces were examined with kinematic measures of walking. The primary interests involved in this study were whether 5- to 7-year old children could adapt their walking to imposed timing constraints, and if so, how their strategies would compare to that of adults. The three main hypotheses were that five- to 7-year olds would: 1) have the ability to modify their walking in response to the audio metronome, 2) have more difficulty meeting imposed timing constraints in comparison to adults, and 3) benefit from practice walking to the audio metronome paces.

## Method

### Ethics Statement

The study and consent procedures were approved by the Boston University Institutional Review Board and conformed to the Declaration of Helsinki. Informed written and verbal consent was obtained from all participants before testing began; caregivers provided written consent on behalf of their children and adult participants provided written consent for themselves.

### Participants

A total of 77 children and adults participated in this study: 57 5- to 7-year-old children (18 5-, 17 6-, and 22 7-year-olds) and 20 adults (*M* age = 23.61 years old, *SD* = 2.14). Age groups were balanced for gender. Inclusion criteria were that children be between 5 and 7 years old, typically developing, born at term, and absent of any physical impairments that precluded independent walking. Most children were Caucasian. No parents reported that their children had experience walking to a metronome beat. Data from one child were not analyzed because the child did not complete the study due to lost interest. Families were recruited via purchased mailing lists and received small souvenirs as thanks for their participation.

Adults were recruited via flyers posted on the Boston University campus and by word of mouth. Most adults were Caucasian. All reported having no injuries or impairments that impeded walking. None had walked to a metronome beat in the past.

### Footfall and Video Recordings

Participants’ responses to the metronome beats during walking were analyzed with footfall and video recordings. A pressure sensitive gait carpet (6.1-m long x 0.89 m wide) measured the distance and timing of each step (GAITRite Inc., Clifton, New Jersey, http://www.gaitrite.com) at a spatial resolution of 1.27 cm and a temporal resolution of 120 Hz. Using the GAITRite software, spatial and temporal parameters were computed with the x- and y-coordinates of the center of pressure for the heels and balls of the foot and first and last times of participants’ foot contacts respectively.

Participants were filmed with three camera views combined into one video frame. Each view captured unique information about participants’ responses. One static camera videotaped participants from the front view. A second camera, panned by an experimenter, filmed participants from the side view. A third camera filmed a light attached to the gait carpet that triggered carpet activation. The light was used to synchronize footfall and video recordings.

### Metronome and Walking Conditions

A timing constraint with an audio metronome (Boss DB-90, www.bossus.com) was imposed to manipulate participants’ walking patterns. The metronome played ticking sounds in beats per minute (bpm). Its tempo ranged from 30–250 bpm with an accuracy of ±0.1% and could be adjusted with a rotary dial in 1-bpm increments. A mute button facilitated quick playing and subsequent silencing of the beat at the beginning and end of each trial. The metronome beat was played at two different speeds: slow (30% slower than participants’ cadence) and fast (15% faster than participants’ cadence).

### Procedure

Children and adults were tested in one 45- to 90-minute session. First, the experimenter measured participants’ weight with a digital scale, standing height from crown to heel with a tape measure attached to a wall, and leg length from anterior, superior iliac spine to medial malleolus with a tape measure. Next, participants were tested in six walking conditions: four to the audio metronome and two at a self-selected pace to no metronome beat. Participants were instructed to “go” at an experimenter’s instruction. For children, parents sat at the end of the gait carpet to encourage them to walk, but were told not to give feedback or cues to their children about their walking.

**Fig 1:**
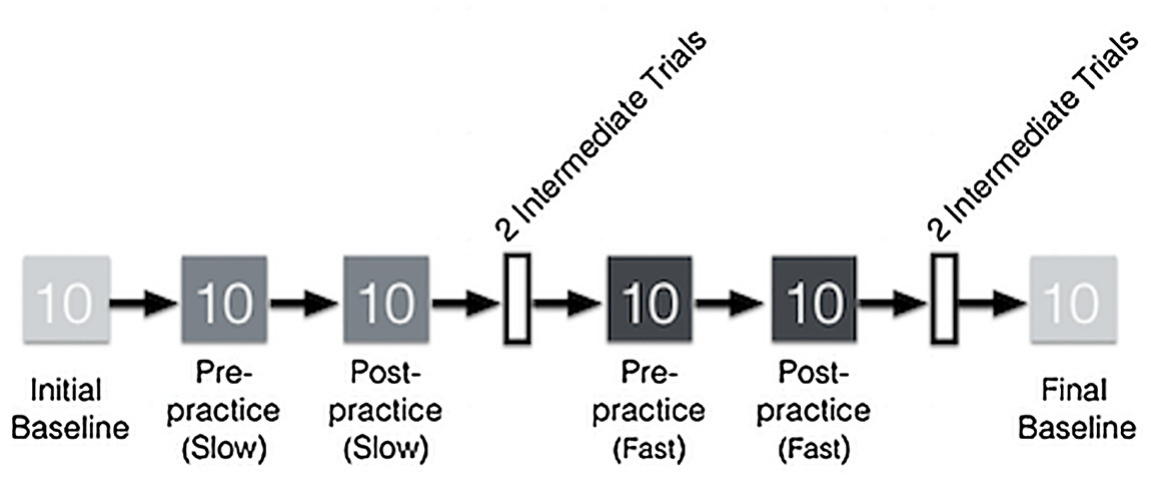
Experimental procedure. The figure shows an example of a session in which the slow metronome pace was played first. Light gray boxes represent initial and final baseline trials. Medium gray are slow metronome paces, and dark gray are fast metronome paces. In between blocks of metronome paces, participants practiced walking to the pace for two minutes. After metronome trials at one pace were complete, participants walked for two intermediate trials at their own pace (white boxes).

Participants walked in metronome and self-paced walking conditions for ten trials in each of the four metronome conditions for a total of 60 trials. Ten trials were selected based on previous research with young children demonstrating that multiple trials are needed for a consistent estimate of walking patterns [10, 11]. Two intermediate trials with no metronome were interspersed between metronome conditions. This was done to allow participants time to recalibrate to their self-selected walking pace prior to introducing the next metronome condition. Therefore, participants walked for a total of 64 trials (Fig 1).

All participants began with an initial baseline condition walking at a self-selected pace to no metronome. Participants’ cadence at the initial baseline was calculated by averaging their cadence at the ten trials. This cadence was then used to create individualized slow and fast metronome paces for each participant. Children and adults were assigned to one of two condition orders: slow, fast or fast, slow. Trials were blocked by metronome pace. After each block of ten metronome trials, participants practiced walking freely around the room for two minutes to the current metronome pace. These two-minute practice periods were introduced to examine if participants’ gait would differ before and after unstructured practice (i.e., walking to the metronome beat around the room). Following the two-minute practice period, while the metronome beat still played, participants were instructed to walk back to the start of the gait carpet and to march in place until hearing the experimenter’s “go” signal. They then completed ten more trials at the same metronome pace. Participants then walked to the two intermediate trials with no metronome prior to beginning the next block of metronome trials.

### Data Processing

The 64 metronome and walking trials were used for analyses. A primary coder scanned for useable walking sequences for footfall analyses using a computerized video coding system, OpenShapa (www.openshapa.org). At least four consecutive walking steps without a disruption in gait (e.g., stopping or tripping) were needed to accurately calculate spatial and temporal parameters. Therefore, a coder tagged trials with the appropriate number of steps and sequences of steps without gait disruptions. Since children were very compliant with completing trials, each useable footfall sequence included 4 to 28 consecutive steps (*M* = 9.91 steps).

Using the x- and y-coordinates and the first and last times that participants’ feet contacted the carpet, the GAITRite software computed spatial and temporal parameters: distance between consecutive steps (step length), walking speed, time with one foot on the ground (single limb support time), time with both feet on the ground (double limb support time), and steps per minute (cadence).

### Data Analyses

SPSS 16.0 software was used to conduct analyses with data presented as averages for all trials in each condition for each participant and standard errors around those means. Baseline walking was characterized by relationships among gait parameters with Pearson’s correlations. Paired t-tests were used to examine participants’ recalibration to their normal walking patterns. The ability to meet the metronome paces, magnitude of deviation from the metronome paces, and carryover effects were tested with repeated measures (RM) analysis of variance (ANOVAs). Tests for the effects of practice reflect comparisons between the average of the ten trials before to the average of the ten trials after two-minutes of practice. Post hoc analyses for RM ANOVAs consisted of pairwise comparisons. To reduce experiment-wise errors because of the multiple tests that were conducted, the Tukey procedure was used for all tests including correlations. Cohen’s *d* is listed after each p-value as a measure of effect sizes for follow up pairwise comparisons [12]. Interpreting effect size is based on the absolute value of Cohen’s *d*. Absolute values of Cohen’s *d* are interpreted as small, medium, or large: absolute values of Cohen’s *d* ≥ 0.2 = small effects, ≥ 0.5 = medium effects, and ≥ 0.8 = large effects.

## Results

### Available Data for Analyses

All participants at each age contributed data in each condition. With the exception of a few conditions, most participants contributed 10 trials. Specifically, all but 18 participants contributed all 10 trials in each condition; sixteen contributed 9 trials in one or two conditions (4 5-year-olds, 4 6-year-olds, 2 7-year-olds, and 6 adults), one 7-year-old contributed 8 trials in one condition, and another 7-year-old contributed 7 trials in a condition. These missed trials were due to equipment failure. Therefore, in total, out of the expected 4,928 trials, 4,907 trials were available for analyses.

### Baseline Walking and Recalibration

**Fig 2:**
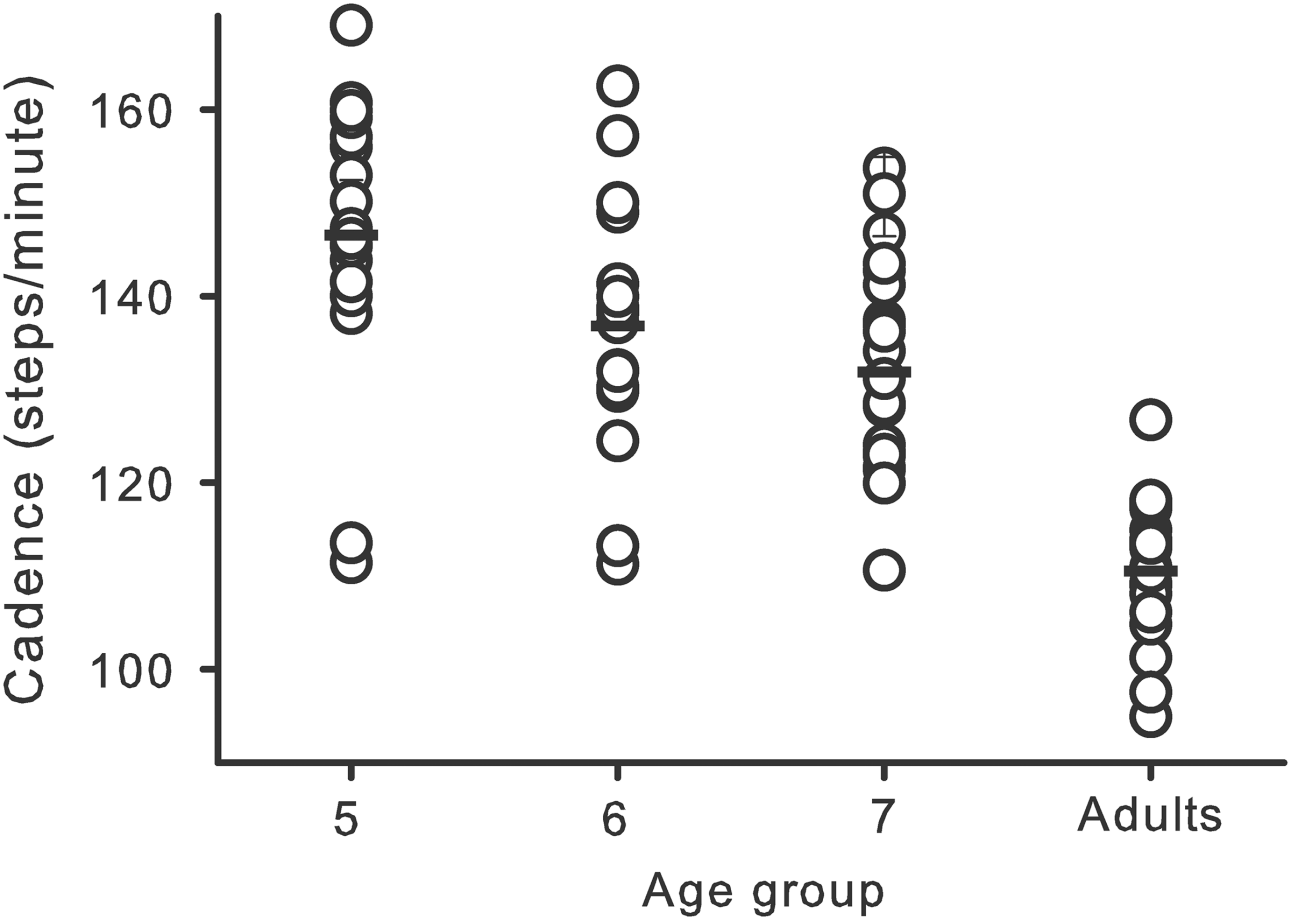
Average initial baseline cadences. Cadence in steps per minute is plotted for each age group. Each circle represents one participant’s average cadence at the initial baseline. Horizontal bars within each age group represent group averages.

The initial baseline condition provided an assessment of participants’ typical walking patterns prior to the introduction of the metronome paces. Therefore, this condition served as a source of comparison for the effects of the metronome paces on participants’ walking patterns. At the initial baseline condition, speed was correlated with step length and single limb support time, which is consistent with previous studies on child and adult walking [3, 4, 13, 14]. Correlation coefficients for speed and step length ranged from .66 to .77 (all *p*s <.01; *d*s from 1.76 to 2.41). For speed × single limb support time, correlation coefficients ranged from-.78 to-.89 (all *p*s <.01; *d*s from -2.49 to -3.90). Speed was also correlated with cadence for 5-year-olds (*r*(18) = .67; *d* = 1.81) and 6-year-olds (*r*(17) = .77; *d* = 2.41) and with double limb support time for the same age groups; *r*(18) = -.82 (*d* = -2.87) and *r*(17) = -.74 (*d* = -2.20) respectively (all *p*s <.01). For the main dependent variable, cadence, each age demonstrated significant correlations with single limb support time (*r*s ranging from-.72 to-.96; *d*s ranging from -2.08 to -6.86) and double limb support time (*r*s ranging from-.56 to-.90); all *p*s <.01. Fig 2 illustrates initial baseline cadence values for each participant.

The final baseline condition allowed for a test of possible residual effects of walking to the metronome paces. Paired t-tests were conducted at each age group to compare all gait parameters at the initial and final baseline conditions. Analyses comparing gait parameters at the initial and final baseline conditions were split for the two metronome pace orders (i.e., whether the slow or fast metronome pace occurred right before the final baseline). When receiving the slow metronome pace last, five-year olds had lower cadences (*t*(9) = 4.10, *p*<.01, *d* = 1.44) and speeds (*t*(9) = 4.18, *p*<.01, *d* = 1.18) and higher single (*t*(9) = -3.63, *p*<.01, *d* = -1.50) and double (*t*(9) = -3.31, *p*<.01, *d* = -1.60) limb support times at the final versus the initial baseline. Table 1 shows average step lengths, velocities, single limb support times, and double limb support times for each age group at the initial and final baseline conditions. Means are split by which metronome pace participants received prior to the final baseline condition.

**Table 1:** Average walking variables for each age group at initial and final baseline conditions.

		Age groups			
		5	6	7	Adults
Step length (cm)	Initial (Slow last)	49.78 (1.96)	52.70 (1.53)	58.19 (2.30)	71.91 (2.84)
	Final (Slow last)	46.73 (1.80)	49.99 (1.03)	60.00 (1.63)	71.67 (2.71)
Step length (cm)	Initial (Fast last)	49.60 (1.27)	50.58 (1.83)	52.16 (1.41)	70.99 (1.73)
	Final (Fast last)	51.33 (1.81)	52.10 (1.62)	54.32 (1.06)	70.06 (1.86)
Single limb support time (msec)	Initial (Slow last)	259.20 (40.82)*	272.50 (43.07)	286.10 (46.97)	289.00 (45.52)
	Final (Slow last)	288.30 (45.53)*	290.50 (44.14)	281.30 (41.12)	302.80 (45.34)
Single limb support time (msec)	Initial (Fast last)	276.10 (46.12)	282.80 (44.46)	280.80 (42.78)	280.60 (40.24)
	Final (Fast last)	262.50 (43.65)	283.10 (45.90)	281.30 (44.66)	284.50 (43.10)
Double limb support time (msec)	Initial (Slow last)	143.10 (43.18)*	159.80 (43.31)	164.80 (44.07)	282.90 (44.46)
	Final (Slow last)	177.20 (44.25)*	218.90 (45.71)	154.80 (44.48)	297.20 (43.90)
Double limb support time (msec)	Initial (Fast last)	158.40 (45.44)	170.50 (44.13)	176.00 (43.22)	258.30 (43.81)
	Final (Fast last)	145.20 (44.07)	167.50 (43.79)	176.50 (44.52)	275.00 (45.73)
Velocity (cm/sec)	Initial (Slow last)	125.32 (14.65)*	123.36 (16.06)	124.72 (14.37)	128.69 (16.17)
	Final (Slow last)	105.26 (16.16)*	107.32 (12.93)	131.62 (14.96)	122.37 (15.75)
Velocity (cm/sec)	Initial (Fast last)	117.79 (16.76)	113.19 (15.93)	116.27 (14.09)	132.61 (13.39)
	Final (Fast last)	125.23 (14.57)	116.02 (14.99)	117.90 (12.96)	128.44 (13.89)
Cadence (steps/minute)	Initial (Slow last)	150.17 (12.45)*	140.23 (15.57)	129.02 (13.74)	107.32 (12.87)
	Final (Slow last)	134.38 (14.70)*	132.42 (16.71)	131.54 (13.73)	102.54 (13.42)
Cadence (steps/minute)	Initial (Fast last)	141.97 (17.23)	133.77 (13.77)	133.85 (13.15)	112.24 (11.93)
	Final (Fast last)	146.50 (13.07)	133.54 (13.71)	130.24 (12.76)	110.18 (12.57)

Means are separated by whether participants received the slow or fast metronome paces prior to the final baseline condition. Standard errors are in parentheses.

* Significant differences (i.e., *p* <.01) are denoted with an asterisk.

### Effects of the Metronome Beat on Walking

Participants’ cadences were influenced by the metronome paces. A 4 (age) × 2 (metronome pace) × 2 (practice) RM ANOVA on cadence revealed main effects for age (*F*(3,73) = 90.89, *p*<.001) and metronome pace (*F*(1,73) = 795.95, *p*<.001). Main effects were qualified by metronome × age (*F*(3,73) = 2.85, *p*<.05) and metronome × practice (*F*(1,73) = 9.06, *p*<.01) interactions. At each age, cadence values were higher at the fast versus the slow metronome pace (all *p*s<.001). At the fast pace, cadence values were slower after practice (*p* <.01). Table 2 shows average cadence values for each age group at the slow and fast metronome paces before and after practice.

**Table 2:** Average cadence at slow and fast metronome paces before (pre) and after (post) practice by each age group. Standard errors are in parentheses.

		Age groups			
		5	6	7	Adults
Cadence (steps/minute)	Slow (Pre)	118.86 (1.57)	104.52 (0.81)	106.83 (0.88)	75.42 (0.15)
	Slow (Post)	120.35 (1.59)	105.63 (0.72)	106.64 (0.82)	75.94 (0.16)
	Fast (Pre)	157.71 (0.93)	154.76 (0.91)	148.35 (1.14)	123.11 (0.26)
	Fast (Post)	154.68 (0.78)	149.94 (0.77)	148.46 (0.99)	123.12 (0.21)

Note significant metronome x age interactions and metronome x practice interactions indicating that all age groups had greater cadences at the fast metronome pace and that cadence decreased at the fast pace after practice.

### Meeting the Metronome Pace

**Fig 3:**
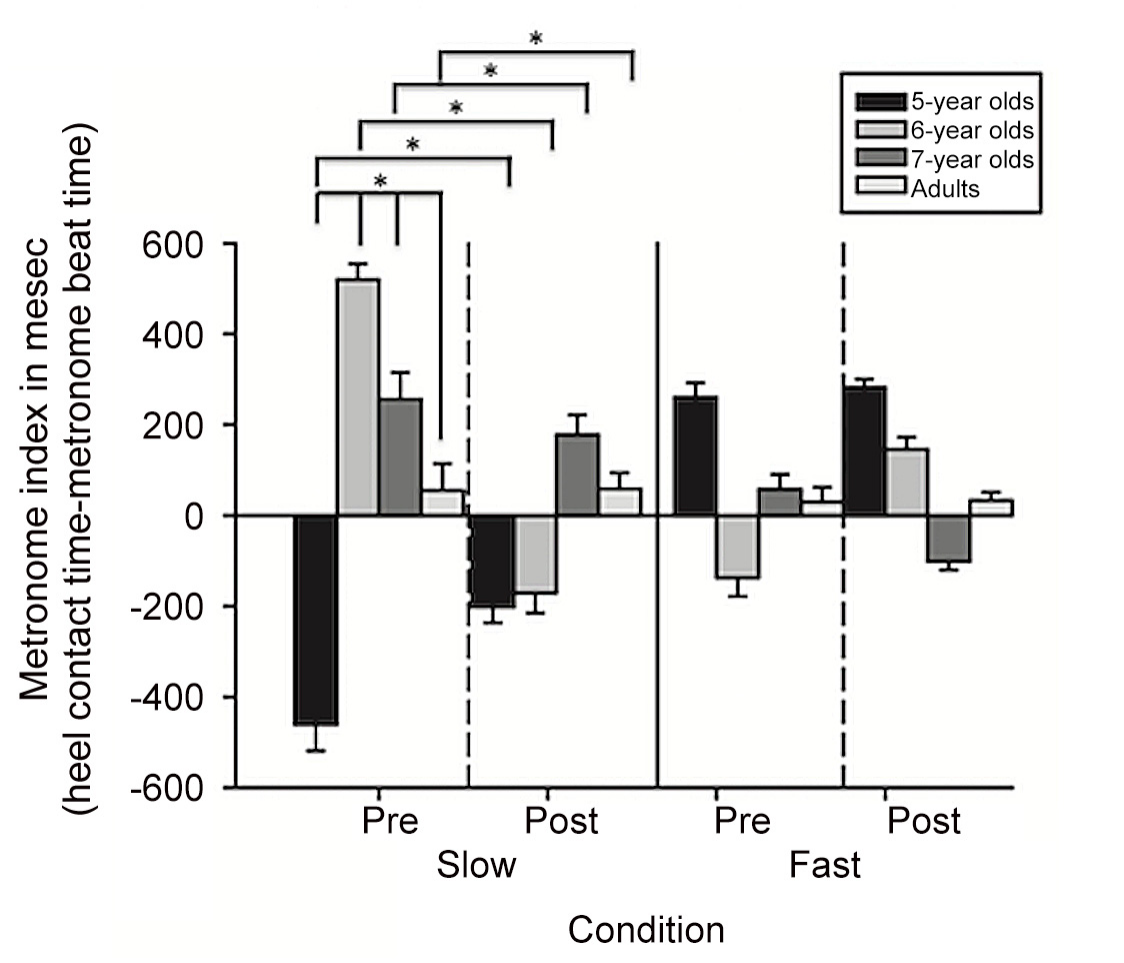
Average metronome index scores. Group averages are plotted for the metronome index (heel contact time—metronome beat time) in milliseconds for 5-year olds (black bars), 6-year olds (medium gray bars), 7-year olds (dark gray bars), and adults (light gray bars). The dashed vertical lines demarcate metronome index means before and after two minutes of practice at each metronome pace. Bars represent standard errors.

A metronome index score (timing of heel contact for each step—timing of the metronome beat) was created to represent how much participants’ walking patterns at each step deviated from the metronome. The metronome index score served as the dependent variable for examining children’s and adults’ ability to meet the metronome paces. A 4 (age) × 2 (metronome pace) × 2 (practice) RM ANOVA tested if 5- to 7-year olds and adults differed in their ability to meet each metronome pace, and if practice influenced the outcome. The RM ANOVA revealed a main effect for age (*F*(3,73) = 7.34, *p*<.01). The RM ANOVA main effect was qualified by a metronome × age interaction (*F*(3,73) = 4.90, *p*<.01). Pearson’s correlations between the metronome index score and gait parameters showed relationships among gait parameters by age. At the slow pace, 5-year olds had negative correlations between the metronome index and single (*r*(18) = -.92; *d* = -4.69) and double (*r*(18) = -.77; *d* = -2.41) limb support time and positive correlations with speed (*r*(18) = .89; *d* = 3.90); all *p*s <.001. Six- and 7-year olds showed a different pattern of results. Both 6- and 7-year olds had negative correlations between the metronome index and single limb support time (*r*(17) = -.80, *d* = -2.67 and *r*(22) = -.71, *d* = -2.02 respectively); all *p*s <.001. However, metronome index and double limb support time was only correlated for 6-year olds (*r*(22) = -.68, *p* <.001, *d-1*.*84*). Speed was correlated with the metronome index at slow paces for 6- and 7-year olds (*r*(17) = .62, *d* = 1.58 and *r*(22) = .64, *d* = 1.67 respectively) and at the fast pace for 6-year olds (*r*(17) = .67, *d* = 1.81); all *p*s <.001. The analyses also revealed a three-way interaction between metronome pace, practice, and age (*F*(3,73) = 5.18, *p*<.01). Five-year olds had the most difficulty meeting the slow metronome pace, but showed improvements after practice (all *p*s <.001; *d*s from 4.60 to 7.19). Six-year-olds, 7-year olds, and adults also showed no differences in their ability to meet the metronome paces before and after practice (all *p*s >.05; *d*s from -1.37 to 3.64) at the slow pace. Fig 3 illustrates metronome index scores in milliseconds for each age at each pace before and after practice.

### Magnitude of Deviation from the Metronome Pace

**Fig 4:**
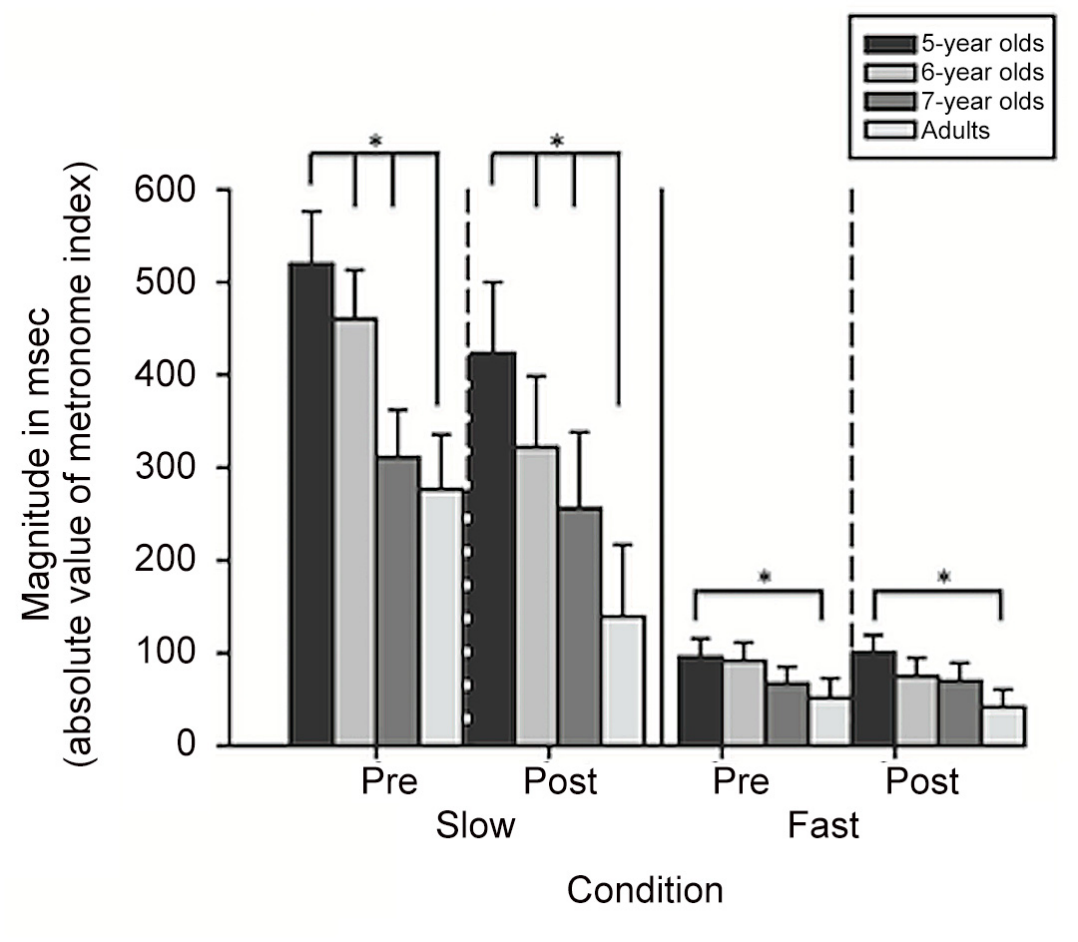
Average magnitude of metronome index scores. The absolute value of metronome index scores are plotted in milliseconds for 5-year olds (black bars), 6-year olds (medium gray bars), 7-year olds (dark gray bars), and adults (light gray bars). The dashed vertical lines demarcate metronome index means before and after two minutes of practice at each metronome pace. Bars represent standard errors.

For an examination of the magnitude of deviation of each step from the metronome, the absolute value of the metronome index score was calculated and used as the dependent variable for a 4 (age) × 2 (metronome pace) × 2 (practice) RM ANOVA. The results showed a metronome × age interaction (*F*(3,73) = 5.15, *p*<.01). Five-year olds showed a difference in how far they deviated from the metronome based on the metronome pace; the magnitude of deviation from the metronome pace was greatest for 5-year-old children compared to other age groups during the slow pace (all *ps* <.001; *d* = 4.22). Fig 4 illustrates the absolute values in milliseconds for the metronome index scores for each age at each pace.

### Carryover Effects

Did the imposition of metronome paces on participants’ walking continue after the metronome paces were removed? To address this question, average cadences were compared during the intermediate trials before the next block of metronome paces (i.e., the average of the first and second intermediate trials) and during average cadence at initial baseline for each metronome condition. A 4 (age) × 2 (metronome pace) × 3 (trial) RM ANOVA confirmed main effects for age (*F*(3,73) = 43.80, *p*<.001) and metronome pace (*F*(1,73) = 49.09, *p*<.001). Five-year olds had higher cadences than the other age groups (all *ps*<.001). Cadence values were higher during initial baseline compared to the average cadence after the slow metronome pace (*p*<.001). Table 3 shows carryover over effects for each group at each metronome pace.

**Table 3:** Average cadence at initial baseline and at the intermediate baseline trials following slow and fast metronome paces by each age group. Standard errors are in parentheses.

		Age groups			
		5	6	7	Adults
Cadence (steps/minute)	Initial baseline	153.75 (3.56)	140.31 (5.37)	130.22 (3.92)	109.61 (1.96)
	Intermediate baseline trial (after slow)	132.09 (4.43)	120.32 (2.62)	119.90 (2.71)	100.72 (2.09)
	Intermediate baseline trial (after fast)	147.03 (3.22)	133.90 (3.24)	128.48 (3.63)	112.72 (1.31)

Standard errors are in parentheses.

## Discussion

School-aged children engage in activities that necessitate changing their walking patterns to meet constraints. Although previous research demonstrates that children’s walking (i.e., at 5- to 7-years old) is similar to that of adults in the absence of imposed constraints, little is known about how their ability to adapt walking compares to adults during this period of time. Would 5- to 7-year-old children modify their walking to meet imposed constraints, and if so, how would their modifications compare to adults?

The present study investigated 5- to 7-year-olds’ and adults’ responses to timing constraints. Footfall recordings were measured as children and adults walked to slow and fast metronome paces that were 30% slower and 15% faster than their average cadences. Below, differences among children and between children and adults are discussed with respect to meeting the metronome paces, task difficulty, and practice and carryover effects. These differences elucidate developmental aspects of adapting to change.

### Meeting the Metronome Pace

Similar to adults [15], the 5- to 7-year olds in this study demonstrated the ability to modify their gait in an attempt to meet timing constraints. Given that changes in metronome timing required children to perceive the task demand and to respond appropriately to each metronome pace, it is significant that children could alter their walking. Five- to 7-year olds’ ability to deviate from their usual walking patterns in response to the metronome shows that they can use their skill in walking to make a change in their responses.

Although children could alter their walking patterns, apparent differences existed in their ability to actually meet the metronome paces in comparison to adults. The findings match the hypothesis; five- to 7-year olds demonstrated the ability to modify their walking, but had difficulty meeting the timing constraint. In comparison to the 5- to 7-year olds, adults did a better job of meeting the timing constraint. This suggests that children do not yet have the capacity to meet timing constraints as accurately as adults. Group patterns emerged in which younger children had more difficulty meeting constraints. Specifically, 5-year-old children demonstrated the most difficulty and had metronome index scores that deviated most from the metronome paces. The 5-year olds in the present study showed that they had enough walking skill to modify their steps in response to the metronome, but had trouble meeting the timing constraint. Therefore, young children may have walking skill similar to adults’ walking skill, but only when walking at self-selected paces. Walking on flat ground at a self-selected pace does not seem to be the same as altering walking to meet a constraint [16, 17]. Even among children with differing physical abilities, comparisons in walking in the absence of constraints can mask differences in movement abilities. Kinematic and kinetic measures during flat ground walking at self-selected paces sometimes reveal no differences between children classified in various weight categories, namely normal weight, overweight, or obese according to body mass index scores (BMI) [18]. However, when asked to cross obstacles of different heights, differences among children surface. Compared to children with normal weight BMI scores, children with overweight and obese BMI scores have more difficulty with motor planning [17] and demonstrate motor strategies [16] and motor skills [19] that would predispose them to falling.

### Task Difficulty

Task difficulty, walking to slow and fast metronome paces, influenced children’s walking. Two possibilities were entertained. One is that walking at a slow pace could be more challenging due to increased balance constraints—standing on one leg until walkers hear the beat that cues the leg in the air to contact the ground. Another is that walking at a fast pace leaves little time in between steps to fix errors if walkers are off beat. The findings showed that walking at the slow pace proved to be more challenging, especially for children. Although some tasks are easy for experienced individuals to accomplish, difficult tasks may require novices to allocate additional attentional resources while participating in the task. For children, walking at a slow pace may have introduced the need to increase attention to simultaneously listen to the beat and match their walking to the beat [19, 20]. In other studies, children demonstrate similar motor responses when completing a task that is challenging for them. They alter their motor patterns, but sacrifice accuracy as a consequence. For example, children at 4- to 6-years old take shorter steps when carrying an empty box but exhibit less accuracy by tilting the box compared to 7- to 12-year olds [19]. Therefore, difficult tasks may thwart attempts to meet constraints despite being a skilled walker.

Walking at a slow pace could have increased balance requirements for children. For 5- and 6-year olds, high metronome index scores (i.e., more deviations from the metronome) were correlated with less time with their feet on the ground and faster walking speeds. One of the most challenging tasks for novice walkers is to maintain balance on one leg as they walk [3, 21–23]. This difficulty is reflected in less time with one foot on the ground and more time with both feet on the ground as well as slower walking speed. The 5-year olds in this study bear similarities to novice walkers because of difficulty walking at a slow pace. However, because they are more skilled then novice walkers, they also have more strategies at their disposal to compensate for difficulty adapting such as increasing walking speed. Increasing speed minimizes the amount of time spent on what was probably the most challenging part of walking slowly: balancing on one leg long enough to wait for the next beat. Although meeting constraints was challenging for younger children, they had the wherewithal to compensate by altering their walking patterns in response to the imposed timing constraint.

### Practice and Carryover Effects

An expected finding is that children had more difficulty than adults meeting metronome paces. Another anticipated finding is that practice helped children to improve, but only at the slow pace. Several explanations are plausible. First, participants were not provided with specific practice walking back and forth on a straight path to the metronome paces. Instead, they were free to move about the room as they liked as long as their steps matched the metronome beat. Improvements in motor skill do occur without specific practice. For instance, with just everyday walking experience, infants learn to modify their steps to walk down slopes of varying degrees [10]. General, everyday walking experience allows children to amass many steps and opportunities to practice adapting their walking under multiple conditions. In the same study, infants who received specific practice on slopes performed better than those who only received everyday experience [10]. However, specific practice may be needed for shorter bouts of practice to boost performance. Also, improvements in adaptation may require specific practice in particular contexts (e.g., structured practice walking to a fast metronome pace). A limitation of the present study includes not examining gait adaptation during the two-minute practice period. However, the purpose of introducing the two-minute practice period was to study its effect on gait adaptation before and after practice. It would be beneficial to quantify gait adaptation during unstructured practice and to compare it to structured practice in future investigations. Second, the task in this study did not involve providing feedback to participants about deviating from the metronome paces. Although practice paired with feedback may have resulted in better performance, the absence of feedback allowed for an observation of children’s and adults’ spontaneous responses to timing constraints. Responses unencumbered by feedback are more similar to how children and adults might respond to everyday situations.

Carryover effects for walking to the metronome only occurred for children, but not for adults. Traditional adaptation studies require recovering from a perturbation, which disrupts normal actions [24, 25]. Children and adults show differences in how they adapt their walking patterns and in how quickly their walking patterns return to normal once the perturbation is removed. For example, when the length of one leg is increased with a platform, adults limp. Once the platform is removed, adults immediately return to their normal walking patterns. In contrast, 14-month-old walkers limp to a lesser extent and require more time to return to their normal walking patterns after removal of the platform [11]. The findings for carryover effects in this study are similar possibly because of a desire to return to normalcy. Compared to children, adults may have more of an ability to immediately return to their normal walking patterns, which would be more energy efficient.

In summary, the present study offers a paradigm for examining children’s ability to modify their walking to meet constraints. Future work is needed to investigate the trajectory of change in walking adaptation among early school-aged children to examine when and how they improve their ability to modify their walking patterns in response to imposed constraints.
